# Clinical and genetic characterization of congenital disorders of glycosylation in 20 Chinese patients

**DOI:** 10.1186/s13023-025-04075-7

**Published:** 2025-12-23

**Authors:** Peiwei Zhao, Li Tan, Qingjie Meng, Lei Zhang, Yufeng Huang, Xiankai Zhang, Yanqiu Hu, Shiqiong Zhou, Xuelian He

**Affiliations:** 1 Genetics and Precision Medical Center, Wuhan Children’s Hospital (Wuhan Maternal and Child Healthcare Hospital), Tongji Medical College, Huazhong University of Science & Technology, Wuhan, 430016 China; 2 Clinical Laboratory, Wuhan Children’s Hospital (Wuhan Maternal and Child Healthcare Hospital), Tongji Medical College, Huazhong University of Science & Technology, Wuhan, 430016 China; 3 Department of Child Health, Wuhan Children’s Hospital (Wuhan Maternal and Child Healthcare Hospital), Tongji Medical College, Huazhong University of Science & Technology, Wuhan, 430016 China

**Keywords:** Congenital disorders of glycosylation, Genetic variants, Genotype-phenotype correlation, Whole-exome sequencing, Novel variants

## Abstract

**Background:**

Congenital disorders of glycosylation (CDG) are a complex and heterogeneous family of rare metabolic diseases that affect protein and lipid glycosylation and glycosylphosphatidylinositol synthesis. These disorders can affect multiple organs, leading to a broad spectrum of symptoms that vary among different CDG subtypes and between individuals with same type of CDG. This study aimed to investigate the genetic variants, molecular etiologies, and clinical features of 20 Chinese patients diagnosed with CDG.

**Results:**

Using whole-exome sequencing (WES), functional prediction tools, Sanger sequencing, and segregation analysis, we identified variants in several genes: *ALG2* (3 patients), *DPM2* (3 patients), *PMM2* (3 patients), and *ALG13* (2 patients). Additionally, variants in *COG5*, *COG6*, *MOGS*, *DPM3*, *ALG1*, *ALG3*, *ALG11*, *SSR4* and *SLC35A2* each were observed in single case. In total, 28 distinct variants were identified, 11 of which were previously unreported. Genotype-phenotype correlations revealed notable findings: variants in the N-terminus of *ALG2* before the intramembrane domain were associated with congenital myasthenic syndromes (CMS), whereas those in the C-terminus caused ALG2-CDG; DPM2-CDG patients with variants in transmembrane region 1 exhibited more severe phenotypes; male patients with hemizygous variants in *SLC35A2* demonstrated milder phenotypes compared to those with mosaic variants.

**Conclusions:**

This findings expand the spectrum of known clinical presentations and genetic variants in CDG, and establish possible genotype-phenotype correlations of several pathogenic genes, emphasizing the need for functional studies to unravel the underlying mechanisms.

## Background

Glycosylation is the predominant form of post-translational modification for proteins and lipids, and glycosylationcan be classified into N-glycosylation, O-glycosylation, glycosphingolipid and glycosylphosphatidylinositol (GPI)-anchor glycosylation, according to the attached amino acids or residues. N-linked glycosylation refers to the covalent attachment of a glycan to nitrogen atom of asparagine residues whileas O-linked glycosylation linked to the hydroxyl group of side chains from serine or threonine residues [1]. The N-glycans are initially synthesized as a branched structure on a lipid anchor in the endoplasmic reticulum (ER), and are critically involved in the processes such as protein folding, trafficking, and signal transduction. Therefore, glycosylationcan plays an important role in various physiological phenomena such as development, immune recognition, and neurogenesis [1, 2].

Congenital disorders of glycosylation (CDG), are a clinically and genetically heterogeneous group of inherited metabolic disorders caused by defects in glycosylation pathways [3, 4]. Glycosylation is ubiquitous, occurring in every cell and organism, with over half of all cellular proteins undergoing glycosylation. Consequently, CDG often present highly diverse clinical presentations and involve multisystems [5, 6]. Typical clinical features of CDG include neurological and developmental disabilities, such as psychomotor retardation/cognitive impairment, epilepsy, hypotonia, and ataxia. Other systems or organs, including hepatic, gastrointestinal, hormonal, and immune systems as well as the heart, eyes, and skeleton, are usually affected. In addition, the severity of symptoms ranges widely, from perinatal death or miscarriage to mild adult-onset forms [7]. Glycosylation is a multicompartmental process occurring in the ER, with the help of precursors synthesized in the cytoplasm, followed by trimming and maturation in the Golgi apparatus, and the process requires lots of glycosyltransferases and glycosidases [8]. According to the mechanism and location of molecular defects, CDG are grouped into disorders of N-linked glycosylation (i.e. PMM2-CDG, ALG12-CDG, SSR4-CDG), disorders of GPI biosynthesis (i.e. PIGN-CDG, PIGA-CDG), disorders of Golgi transport (i.e. SLC35A2-CDG, SLC35C1-CDG), disorders of Golgi trafficking (i.e. COG6-CDG, COG4-CDG), etc [9]. More than half of CDG are due to defects in the N-glycosylation pathway, the remaining ones affecting O-glycosylation pathway or multiple glycosylation pathways. Serum transferrin and apolipoprotein C-III, can be used as a diagnostic biomarkers, for most N-linked glycosylation disorders and mucin core1 O-glycosylation defects [10]. However, these related approaches are labor-intensive and time-consuming, and are unsuitable for screening for all CDG. In addition, serum glycosylation spontaneously or gradually normalizes over time, which makes the diagnosis more challenging (notably in adult individuals) [6]. Therefore, a timely and comprehensive detection method, next-generation sequencing (NGS), has offered great additional value, and rapidly expanded both the discovery of novel glycosylation-related disorders and the unraveling of many unsolved cases.

With the advent of NGS in clinical practice, the number of identified CDG has exponentially increased, to date, over 180 subtypes of CDG have been identified [11]. However, due to the rarity, novelty, clinical heterogeneity of CDG, patients with this disorder often face significant delays in diagnosis and limited treatment options, resulting in poor outcomes. In this study, we included a total of 20 patients whose clinical features and genetic data were consistent with the diagnosis of CDG by retrospectively analyzing all patients who underwent whole exome sequencing (WES) from August 2018 to March 2025 in Wuhan Children’s Hospital. Among these 20 patients, we identified 28 variants in several genes: *ALG2* (3 cases), *DPM2* (3 cases), *PMM2* (3 cases) and *ALG13* (2 cases), with the remaining patients carrying variants in *COG5*, *COG6*, *MOGS*, *DPM3*, *ALG1*, *ALG3*, *ALG11*, *SSR4* and *SLC35A2*, 11 of which are novel and have not been reported. Our study provide valuable insights into CDG genetic diversity and offer foundational clues for future diagnosis and therapeutic advancements.

## Materials and methods

### Study subjects

As mentioned above, we retrospectively analyzed a total of 4912 patients who were suspected of genetic disorders and underwent WES from August 2018 to March 2025 in Wuhan Children’s Hospital, and identified 20 patients suspected or diagnosed of having CDG, along with their healthy family members. All patients were born into families with healthy parents and exhibited normal at birth. Six families underwent prenatal diagnosis following a confirmed diagnosis. Detailed medical and clinical data, including family history, MRI neuroimaging, electroencephalography (EEG), and other relevant examinations, were collected. The study was approved by the ethics committee of the Institutional Review Board of Wuhan Children’s Hospital, Tongji Medical College, Huazhong University of Science & Technology, with the approval code 2021R060-E1. Written informed consent was secured from all participants whose data are presented individually in accordance with the principles outlined in the Declaration of Helsinki.

### Whole-exome sequencing, copy number variation sequencing and data analysis

WES and subsequent data analysis were conducted with the help of the third party medical laboratory (Chigene Lab, Beijing China). For WES, the target DNA fragments were enriched by hybridization to construct an exome library (IDT The xGen Exome Research Panel v1.0). High-throughput sequencing was performed using an Illumina NovaSeq 6000 sequencer. Single-nucleotide and indel variants were identified using the Genome Analysis Toolkit (GATK). Paired-end alignment was performed using the Burrows-Wheeler Aligner (BWA). Moreover, ANNOVAR software annotated and filtered the variants. SNPs and indels were filtered and screened according to sequence depth and variant quality. The variants were annotated using the OMIM, ClinVar and HGMD databases. Candidate variants were confirmed by Sanger sequencing using self-designed primers in the patients and their parents.

To predict the pathogenicity of the variant, we used several bioinformatics software and tools.

At first, variants were preferentially selected for further analysis and validation with their minor allele frequency (MAF) < 0.01 in the ExAc browser (https://exac.broadinstitute.org) and gnomAD (https://gnomad.broadinstitute.org) database. Then, single nucleotide variants (SNV) and short indel candidates were identified. The SIFT utilities were used to forecast the change in protein structure. Conservation of each amino acid change was calculated using PhyloP2 and MutationTaster (http://www.mutationtaster.org/) algorithms were used to predict the effects of variants on protein function. All variants were named according to the guidelines of the Human Genome Variation Society (http://www.hgvs.org/) and were described and classified based on the standard guidelines of American College of Medical Genetics and Genomics (ACMG) [12]. According to the ACMG guidelines, variants are classified as pathogenic, likely pathogenic (LP) and variant uncertain significance (VUS).

The copy number variation sequencing (CNVseq) methodology employed in this study is a low-coverage whole-genome sequencing (WGS) strategy to achieve comprehensive genomic coverage. This approach enables the detection of large-scale CNVs across the genome.

WES detection has its advantages over single nucleotide variations and small insertion/deletion variations (< 20 bp), however, WES does not cover 100% the exome, thus variations within deep intronic and regulatory regions cannot be detected. In addition, WES is not validated for the detection of structural variations (SVs), including inversions and translocations. Due to the deepth of sequencing, low-level mosaicism (< 10%) may be missed.

### In vitro splicing assay

To investigate the effect on the splicing process of the *COG5* gene, minigene splicing assay was performed based on the pcDNA3.1(+) expression vector. Fragments of *COG5* gene, including exon7-exon10 was amplified and connected to construct minigene vectors. HEK 293T cells were cultured in 6-well plates and transfected with wildtype and mutant plasmid using Lipofectamine 3000 (Invitrogen) according to the manufacturer’s instructions. Cells were harvested 48 h later, RNA was extracted from each group using TRIzol reagent, *COG5* was amplified by PCR using site specific primers and the PCR products were separated by 2% agarose gel electrophoresis and sequenced on ABI 3500DX equipment.

### Genotype-phenotype associations

The novel candidate variants in this study or previously reported variants to cause CDG disease was summarized and reviewed and adapted to the clinical symptoms of each patient. They were also determined using several databases such as Human Gene Mutation Database, PubMed (https://www.ncbi.nlm.nih.gov/pubmed/), and Online Mendelian Inheritance in Man (https://omim.org/) to analysis the association between genotype and phenotype.

## Results

### Overall characteristics and molecular diagnosis

**Fig. 1:**
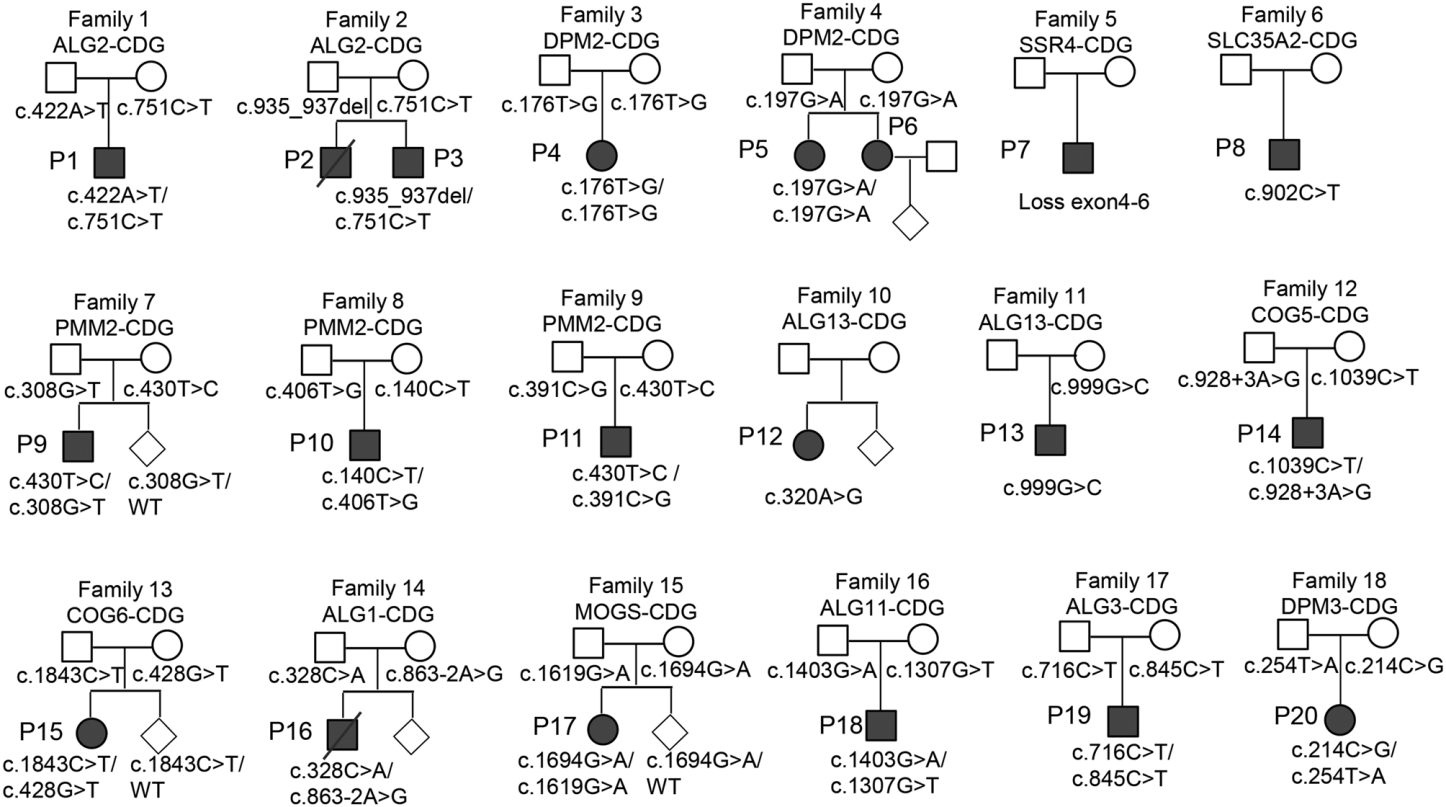
Pedigrees of 18 families (20 patients) with CDG. Squares and circles represent males and females, respectively, while diamonds indicate cases of prenatal diagnosis. A slash through a symbol denotes deceased individuals

**Fig. 2:**
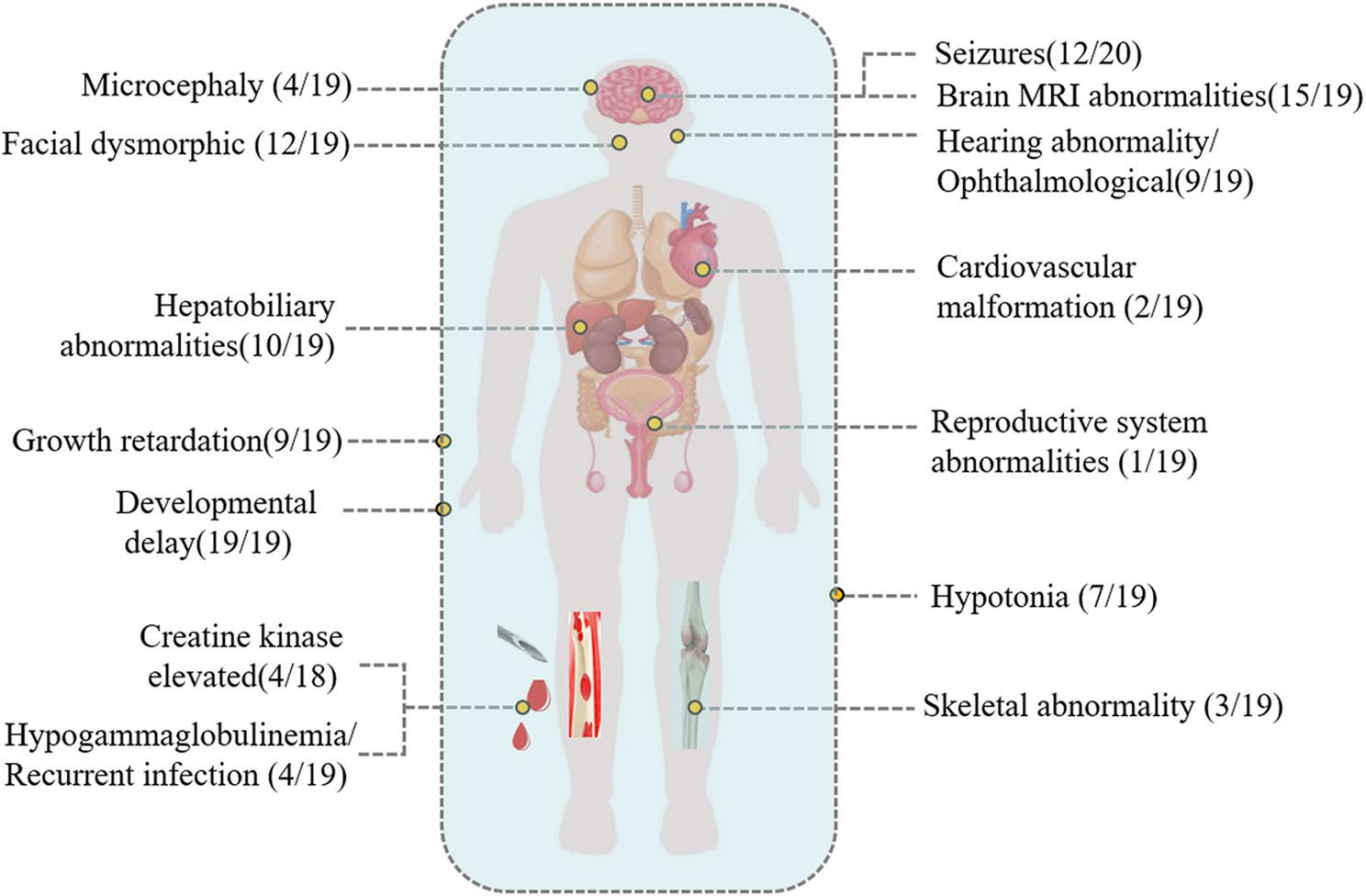
Proportion of individuals presenting with specific symptoms at diagnosis in our cohort

This study included 20 CDG patients (13 males and 7 females) from 18 non-consanguineous families (Fig. 1; Table 1). Clinical symptoms were presented for 20 cases and cataloged using Human Phenotype Ontology (HPO) terms (Table 1). The overall median age of symptom onset was 6.0 months (range: 1.0–48.0 months), and the median age at diagnosis was 1.45 years (range: 4 months to 20 years). The most common symptoms were developmental delay (100%, 19/19), followed by facial dysmorphia (63.2%, 12/19), seizures(60%, 12/20), abnormal liver function (52.6%, 10/19), and hearing or ophthalmological problems (47.4%, 9/19) (Fig. 2).

**Table 1:** Clinical manifestation of patients in this cohort

Family NO.	Patient NO.	Sex	Age of onset	Age of diagnosis	First symptoms	Dysmorphic features	Developmental delay	Growth retardation	Microcephaly	Hypotonia	Recurrent infection	Hepatobiliary abnormalities	Skeletal abnormality	Seizure	Hearing abnormality/ Ophthalmological	Brain MRI	CDG type	Other findings
Family 1	P1	M	3 M	8 M	Epileptic seizures	no	Yes	no	no	no	no	no	no	Infantile spasm	No	Width of cerebral sulcus	CDG Ii ALG2-CDG	serious language delay
Family2	P2	M	4 M	4 M	Epileptic seizures	no	Yes	yes	no	no	no	Elevated in AST, ALT	no	Infantile spasm	No	no	CDG Ii ALG2-CDG	serious language delay
	P3	M	3 M	7 M	Epileptic seizures	NA	NA	NA	NA	NA	NA	NA	NA	Treatment-resistant epilepsy	NA	NA	CDG Ii ALG2-CDG	Died at 7 M
Family 3	P4	F	4Y	11Y	Abnormal walking posture	Bow shaped eyebrows, strabismus	Mild language, motor delay	No	No	hypertonia	No	No	No	Abnormal EEG, mild	Strabismus	NO	CDG Iu DPM2-CDG	CK increased; Inguinal hernia
Family 4	P5	F	NA	20Y	Mild intellectual disability	Full moon face, broad nose	Yes	No	No	No	No	No	No	No	no	NA	CDG Iu DPM2-CDG	CK increased
	P6	F	6 M	8Y	Epileptic seizures	Abnormal eyebrows, eye distance	Language, motor delay	No	No	No	No	No	No	Yes	No	Enlargement of the ventricular system	CDG Iu DPM2-CDG	CK increased
Family 5	P7	M	1 M	16 M	Global Developmental Delay	Abnormal eye distance; flat nose;	Global Developmental Delay	Yes	No	yes	No	No	No	No	Strabismus	no	CDG Ir SSR4-CDG	Intrauterine growth retardation
Family 6	P8	M	18 M	35 M	Language, motor delay	Micrognathia, Downward tilt of eyes	Language, motor delay	Short stature(<-3sd)	Yes	No	No	Elevated in AST	Pectus carinatum	Abnormal EEG	Hyperopia	Delayed myelination of the brain	CDG IIm SLC35A2-CDG	no
Family 7	P9	M	6 M	1Y	Developmental delay	Strabismus; Abnormal eyebrows	Yes	Height weight(<-3SD)	no	hypertonia	No	Elevated in AST, ALT	no	YES	Strabismus, Nystagmus	Cerebellar atrophy	CDG Ia PMM2-CDG	decreased myodynamia; Decreased antithrombin
Family 8	P10	M	3 M	6 M	Developmental delay	No	Language, motor delay	NA	No	No	Yes	Elevated in AST, ALT	No	Abnormal EEG; Febrile convulsion	Strabismus	Cerebellar atrophy	CDG Ia PMM2-CDG	activated partial thromboplastin time increased
Family 9	P11	M	17 M	19 M	Developmental delay	No	Language, motor delay	NA	No	No	No	Elevated in AST, ALT	No	No	No	Cerebellar atrophy	CDG Ia PMM2-CDG	activated partial thromboplastin time increased
Family 10	P12	F	6 M	14 M	Epileptic seizures	No	Yes	No	No	No	Yes	No	No	Infantile spasm	No	Widening of bilateral lateral fissuresxtra cranial space	ALG13-CDG	no
Family 11	P13	M	17 M	19 M	Epileptic seizures	No	Language, motor delay	No	no	No	No	Increased r-GT	No	Infantile spasm	No	Right choroidal cyst	ALG13-CDG	no
Family 12	P14	M	7 M	3Y8M	Motor delay	Abnormal eye distance; flat nose	Yes	Yes	no	Mild	NA	Elevated in AST, ALT	Scoliosis	NA	Myopia nystagmus	Cerebellar atrophy	CDG IIi COG5-CDG	Small penis and testicles
Family 13	P15	F	8 M	20 M	Psychomotor delay	Narrow forehead, hypertelorism, bulbous nose	Yes	Yes	Yes	NA	Yes	Cholestasis, abnormal liver function	Adducted thumbs and camptodactyly	Abnormal EEG	No	Lesions of bilateral temporal, occipital, and parietal lobes	CDG IIl COG6-CDG	atrial septal defect
Family 14	P16	M	4 M	1Y	Epileptic seizures	Micrognathia; abnormal eye distance	Yes	Yes	Yes	hypertonia	No	Elevated in AST, ALT	No	Treatment-resistant epilepsy	no	Small volume of white matter	CDG Ik ALG1-CDG	Died at 14 M
Family 15	P17	F	1 M	4 M	Epileptic seizures	Full moon face, broad nose, narrow forehead	Yes	Yes	no	Yes	Low levels of IgG, IgA, IgM	Hepatomegaly, elevated hepatic enzymes	no	Infantile spasm	Low vision, small palpebral fissure	Thin corpus callosum; wide cerebral sulcus	CDG IIb MOGS-CDG	Heart PFO/ASD
Family 16	P18	M	3 M	4 M	Epileptic seizures	Narrow forehead, downturned corners of the mouth, and a bulbous nose	Yes	Yes	Yes	hypertonia	No	no	no	epileptic encephalopathy	Hearing loss	reduced frontal volume	CDG Ip ALG11-CDG	absent speech
Family 17	P19	M	NA	11Y	Language delay; unilateral hearing loss	Congenital facial paralysis, low-set eyes	Language, motor delay	No	No	NA	No	No	No	yes	Amblyopia in both eyes, hearing loss	Enlargement of the ventricular system	CDG Id ALG3-CDG	unilateral facial palsy
Family 18	P20	F	6 M	7Y	Developmental delay; hypotonia	No	Yes	No	No	yes	No	No	No	Yes	no	cerebral white matter demyelination	DPM3-CDG	CK increased

NA: Not Available

By using WES, we identified 28 disease-causing variants in 13 genes among the 20 patients. Among these variants, 17 previously reported and 11 novel variants. A summary of detected variants is provided in Table 2.

**Table 2:** Variant information of disease-causing genes was detected in the study

Family	No.	Gene and transcript	Variant	Protein change	rs ID	SIFT	Polyphen	CADD score	REVEL	gnomAD	ACMG class	ACMG criteria	References
Family 1	P1	ALG2 NM_033087	c.422 A > T	p.D141V	NA	damaging	0.999	28.3	0.945	6.20E-07	LP	PM2-supporting, PP3-strong, PP4	This work
			c.751 C > T	p.R251C	rs568029420	tolerated	0.973	25.7	0.491	0.000002	LP	PM2-supporting, PP4,PM3,PP1,PP5	30,945,278
Family 2	P2	ALG2 NM_033087	c.935_937del	p.L312del	rs757232575	NA	NA	NA	NA	NA	LP	PM2-supporting, PP4,PM4-supporting, PP1,PM3	30,945,278
			c.751 C > T	p.R251C	rs568029420	tolerated	0.973	25.7	0.491	0.000002	LP	PM2-supporting, PP4,PM3,PP1,PP5	30,945,278
	P3	ALG2 NM_033087	c.935_937del	p.L312del	rs757232575	NA	NA	NA	NA	NA	LP	PM2-supporting, PP4,PM4-supporting, PP1,PM3	30,945,278
			c.751 C > T	p.R251C	rs568029420	tolerated	0.973	25.7	0.491	0.000002	LP	PM2-supporting, PP4,PM3,PP1,PP5	30,945,278
Family 3	P4	DPM2 NM_003863	c.176T > G (homo)	p.L59R	NA	damaging	0.999	24.8	0.656	NA	VUS	PM2-supporting, PP3	This work
Family 4	P5	DPM2 NM_003863	c.197G > A (homo)	p.G66E	NA	damaging	0.999	32	0.837	NA	LP	PM2-supporting, PP3-moderate, PS3,PP4	37,152,991
	P6	DPM2 NM_003863	c.197G > A (homo)	p.G66E	NA	damaging	0.999	32	0.837	NA	LP	PM2-supporting, PP3-moderate, PS3,PP4	37,152,991
Family 5	P7	SSR4 NM_006280	Loss exon4-6	NA	NA	NA	NA	NA	NA	NA	P	PVS1, PM2-supporting, PP4	This work
Family 6	P8	SLC35A2 NM_005660	c.902 C > T	p.T301I	NA	damaging	0.887	23.4	0.448	0	VUS	PM2-supporting	This work
Family 7	P9	PMM2 NM_000303	c.430T > C	p.F144L	rs150719105	damaging	0.988	24.9	0.956	0.0002	P	PM2-supporting, PS1, PM5, PM3_Strong, PP3_Strong	34,671,977
			c.308G > T	p.C103F	NA	damaging	0.999	32	0.977	NA	LP	PM3,PM2-supporting, PP3-strong, PP4	11,058,895
Family 8	P10	PMM2 NM_000303	c.140 C > T	p.S47L	rs138306798	damaging	0.995	34	0.888	0.00004	LP	PM3,PM2-supporting, PP3-modetare, PP4	33,413,482
			c.406T > G	p.C136G	NA	damaging	0.983	26.6	0.966	NA	LP	PM2-supporting, PM3,PP3-strong	This work
Family 9	P11	PMM2 NM_000303	c.430T > C	p.F144L	rs150719105	damaging	0.988	24.9	0.956	0.0002	P	PM2-supporting, PS1, PM5, PM3_VeryStrong, PP3_Strong	34,671,977
			c.391 C > G	p.P131A	rs1274547742	damaging	0.975	26.2	0.905	NA	LP	PM3.PM2-supporting, PP3-moderate, PM5	9,140,401
Family 10	P12	ALG13 NM_001099922	c.320 A > G	p.N107S	rs398122394	damaging	0.858	24.2	0.277	NA	LP	PS1,PM5,PM2-supporting, PP4	36,930,724
Family 11	P13	ALG13 NM_001257231	c.999G > C	p.K333N	NA	damaging	0.553	25	0.06	NA	VUS	PM3.PM2-supporting	This work
Family 12	P14	COG5 NM_001161520	c.1039 C > T	p.Q347*	NA	NA	NA	41	NA	NA	P	PVS1, PM2-supporting, PP4	This work
			c.928 + 3 A > G	NA	NA	NA	NA	NA	NA	0.00001	LP	PS3,PM2-supporting, PM3,PP4	This work
Family 13	P15	COG6 NM_020751	c.1843 C > T	p.Q615*	NA	NA	NA	47	NA	NA	P	PVS1, PM2-supporting, PP4	34,331,832
			c.428G > T	p.S143I	NA	damaging	0.269	24	0.119	NA	VUS	PM2-supporting, PM3, PP4	34,331,832
Family 14	P16	ALG1 NM_019109	c.328 C > A	p.Q110K	NA	damaging	0.939	24.9	0.75	NA	LP	PM2-supporting, PP3,PM3,PP4,PP5	35,715,422
			c.863–2 A > G	NA	NA	NA	NA	NA	NA	0.00003	P	PVS1, PM2-supporting, PP4	This work
Family 15	P17	MOGS NM_006302	c.1694G > A	p.R565Q	rs777861500	damaging	0.998	7.527	0.255	0.000019	LP	PM2-supporting, PP4,PS3	31,925,597
			c.1619G > A	p.R540H	NA	damaging	0.999	32	0.446	0.000008	LP	PM2-supporting, PP4,PS3	31,925,597
Family 16	P18	ALG11 NM_001004127	c.1403G > A	p.R468H	rs1204420316	damaging	1	34	0.846	0.000008	P	PM2-supporting, PM5,PS3,PP4,PP3-modetare	39,260,222
			c.1307G > T	p.G436V	NA	damaging	1	28.7	0.917	NA	P	PM3,PM2-supporting, PP3-modetare, PP4,PS3	39,260,222
Family 17	P19	ALG3 NM_005787	c.716 C > T	p.A239V	NA	damaging	1	33	0.8	0	VUS	PM2-supporting, PP3-moderate, PP4	This work
			c.845 C > T	p.A282V	NA	tolerated	0.386	22.7	0.258	0.0009	VUS	PM2-supporting, PP3-moderate, PP4	This work
Family 18	P20	DPM3 NM_018973	c.124 C > G	p.P42A	rs745692004	damaging	1	29.6	0.931	0.000007	LP	PM3.PM2-supporting, PP3-moderate, PP4	31,469,168
			c.254T > A	p.L85*	rs121908155	NA	NA	42	NA	NA	P	PVS1, PM2-supporting, PP4	31,469,168

The patients were followed from 6 months to 5 years in outpatient of Wuhan children’s hospital. During this time, two patients died due to recurrent intractable epilepsy associated with their diseases (P3 and P16). Prenatal diagnosis was performed in six families (F4, F7, F10, F13, F14 and F15) using amniotic fluid cells.

### Patients with variants in the *ALG2* gene

Variants in the *ALG2* gene (NM_003087) are associated with ALG2-CDG, also known as CDG Ii (OMIM#607906), which is characterized by neurological symptoms such as convulsive syndrome, epilepsy, axial hypotonia, mental and motor regression [13]. Additionally, *ALG2* variants can cause congenital myasthenic syndrome type 14 (ALG2-CMS, OMIM#616228), which presents within the first decade of life, progresses slowly [14].

**Fig. 3:**
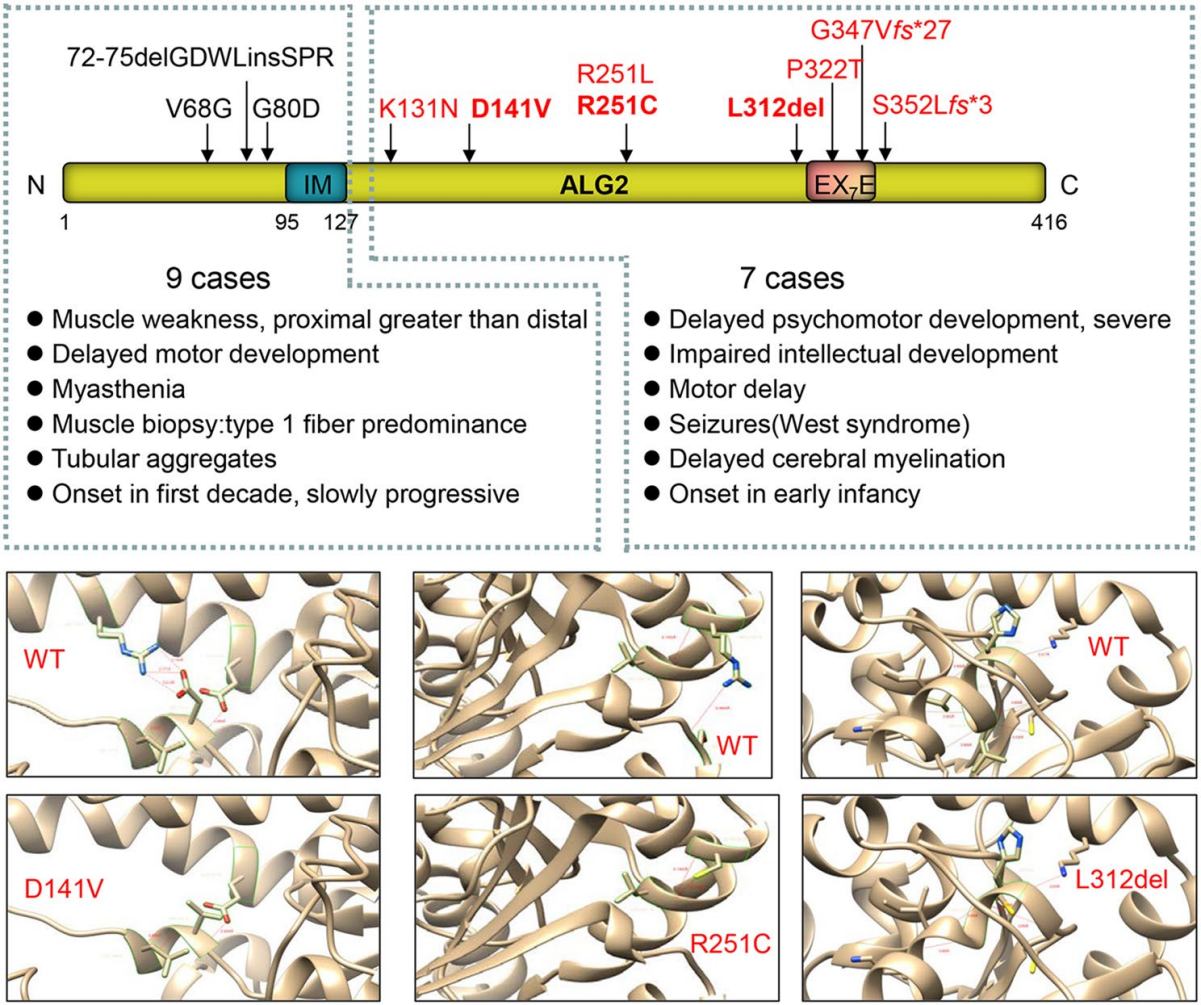
(**A**) Distribution of *ALG2* variants and genotype-phenotype analysis. Variants associated with CDG are highlighted in red, while those linked to CMS are marked in black. IM: intramembrane. (**B**) Predicted effects of *ALG2* variants identified in this study on protein stability using a three-dimensional structural model. WT: wild type

In this study, three patients (P1, P2 and P3) from 2 families with compound heterozygous *ALG2* variants were included. The initial symptom of these 3 patients was epileptic seizures. All of them exhibited seizures, mental and developmental regression, severe speech defects, and abnormal liver function. Genetic analysis identified compound heterozygous variants (c.422 A > T (p.D141V) and c.751 C > T (p.R251C) in P1, and variants (c.751 C > T and c.935_937del (p.L312del)) in P2 and P3. According to standard guidelines of ACMG, c.751 C > T was classified as LP, while c.422 A > T and c.935_937del as LP. The potential effects of these variants were predicted using a three-dimensional structural model, indicating Protein stability being reduced compared to the wild type (lower panel in Fig. 3). Based on typical clinical manifestations and genetic findings, these patients were diagnosed with ALG2-CDG.

### Patients with variants in the DPM2 gene

DPM2-CDG (OMIM#615042) is an extremely rare subtype of CDG. In this study, P4 presented with seizures, followed by neurological and motor developmental delay, and elevated creatine kinase (CK) levels. Genetic analysis identified a homozygous variant c.176T >G (p.L59R) in exon 4 of *DPM2* (NM_003863.4), inherited from heterozygous parents. According to standard guidelines of ACMG, this variant was classified as VUS. Two additional cases, P5 and P6, had been previously reported [15]. Together with our case in this study, only seven cases have been identified [16, 17].

### Patients with variants in SSR4 gene

**Fig. 4:**
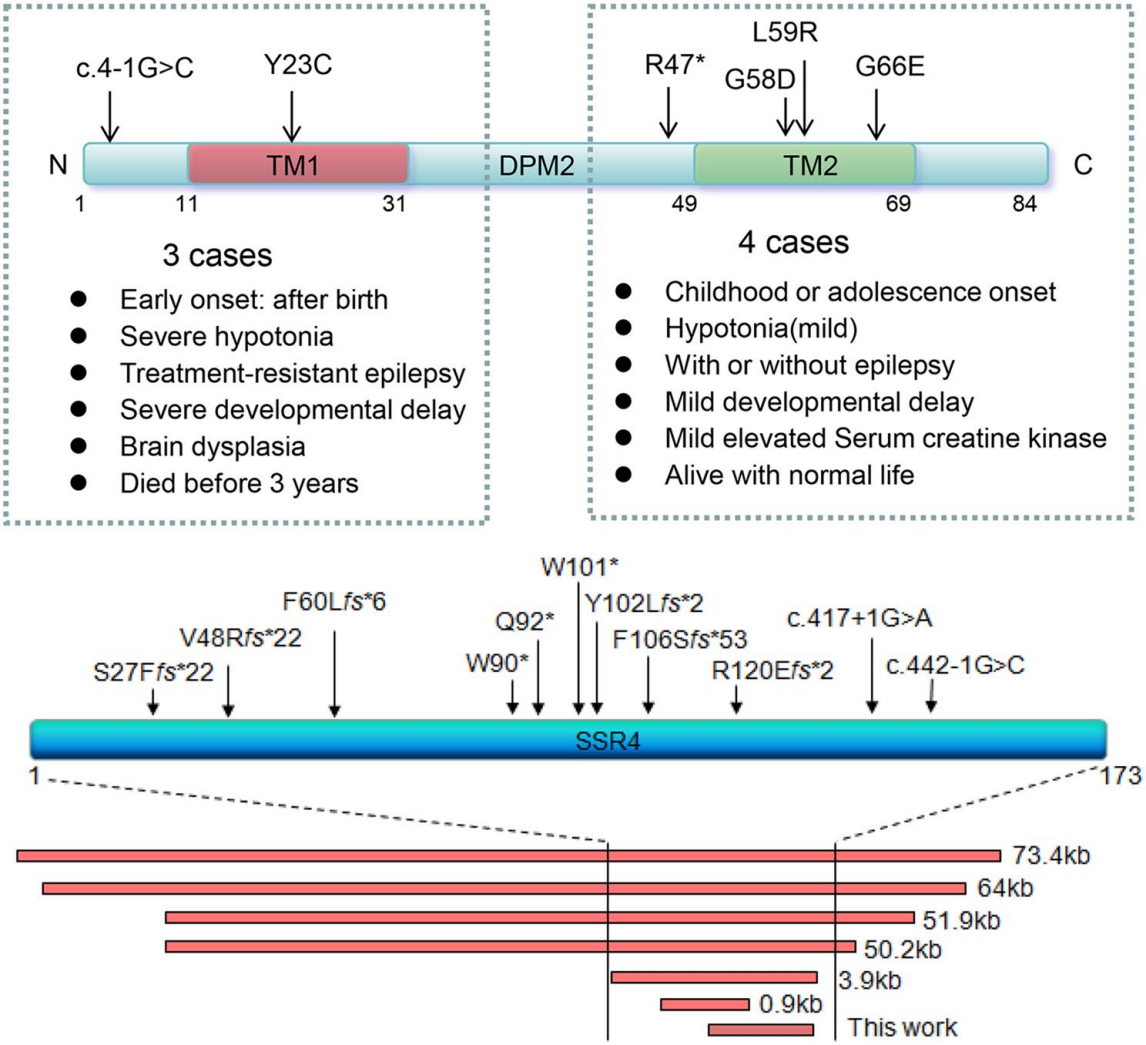
(**A**) Distribution of *DPM2* variants and genotype-phenotype analysis. Patients with variants highlighted in red exhibited milder symptoms, while those with variants in black experienced severe symptoms and died before age 3 years. (**B**) Distribution of *SSR4* variants, including fragment deletions. TM: transmembrane

SSR4-CDG is a rare and relatively mild subtype of CDG (also known as CDG Iy), predominantly affecting males [18]. Key clinical features include developmental delay, speech delay, intellectual disability, muscular hypotonia, microcephaly, skeletal abnormalities, and distinct facial features [19]. In this study, we identified a *de novo* hemizygous deletion spanning exons 4 to 6 of *SSR4* in P7, a 16-month-old boy who had healthy parents, and the deletion was classified as pathogenic. He was born at 39 weeks following a pregnancy complicated by intrauterine growth retardation, and present with global developmental delay, speech delay, cognitive impairment, hypotonia, febrile seizures, feeding difficulty, and mild facial dysmorphisms (large ears, smooth and long philtrum, abnormal eye distance, prominent nose). A literature review revealed 11 point variants and 7 fragment deletions in *SSR4* from 23 SSR4-CDG patients(Fig. 4B). High degree of phenotypic heterogeneity across individuals was found and no clear genotype-phenotype correlation has been established.

### Patients with variants in SLC35A2 gene

SLC35A2-CDG, an X-linked dominant disorder, typically manifests during infancy. The condition is characterized by microcephaly, psychomotor development delay, epileptic seizures, and hypotonia [20]. Male patients usually carry mosaic variants [21]. A hemizygous variant (c.902 C >T, p.T301I) in was *SLC35A2* was identified in P8. He was a 35-month-old boy with short stature (<-3sd), mild language and motor developmental delay, mild facial dysmorphisms, pectus carinatum, and elevated aspartate transaminase (AST) levels. According to the ACMG guidelines and clinical manifestations, this variant was classified as VUS. A literature review identified four male cases with hemizygous variants in *SLC35A2* [22–24], and their clinical features included short stature, abnormal liver function, language delay, intellectual disability, mild facial abnormalities, skeletal deformities and epileptic seizures [23]. In addition, male patients with mosaic *SLC35A2* variants were reported to exhibit more severe symptoms, including epileptic encephalopathy or drug-resistant focal epilepsy [25, 26]. Based on these findings, we propose that male patients with hemizygous *SLC35A2* variants generally present with milder phenotypes compared to those with mosaic variants.

### Patients with variants in the PMM2 gene

PMM2-CDG (CDG Ia, OMIM#212065) is the most common CDG with more than 1000 cases recorded worldwide [27, 28]. Three patients (P9, P10, and P11) presented with developmental delay and hypotonia. They also had abnormal liver functions, evidenced by elevated AST and alanine aminotransferase (ALT). Brain MRI showed cerebellar atrophy and white matter demyelination. Compound heterozygous variants in *PMM2* (NM_000303.3) were identified in all three cases. The variant c.406T >G (p.C136G) in P10 was first reported in this work and classified as LP (PM2-supporting, PM3, PP3-strong) according to ACMG guidelines.

### Patients with variants in the ALG13 gene

ALG13-CDG (OMIM#300884) is a rare X-linked CDG, which affects the N-linked glycosylation pathway [29]. Affected individuals typically exhibit refractory seizures, psychomotor development delay, poor or absent speech, hypotonia, facial dysmorphism, and intellectual disability [30]. Epileptic spasm is a common presenting symptom of ALG13-CDG.

Two unrelated patients (P12 and P13) with infantile spasms associated with hypsarrhythmia on EEG as the initial symptom was identified (Table 1). Both patients currently exhibit delayed language and motor development. In addition, P16 has the characteristic of recurrent infections. Brain MRI showed a right choroidalcyst and widening of bilateral lateral fissures intracranial space. A *de novo* heterozygous variant c.320 A > G (p.N107S) in *ALG13* (NM_001099922.3), a known hotspot pathogenic variant, was identified in P12. In P13, a hemizygous variant (c.999G > C, p.K333N) was found and classified as a VUS, inherited from the patient’s unaffected mother.

### Patients with variants in COG5 and COG6 genes

The COG (conserved oligomeric Golgi) complex is composed of 8 subunits, including lobe A (from subunit COG1 to COG4) and lobe B (from subunit COG5 to COG8) [31]. Genetic defect in all subunits disrupts the function of the COG complex and have been reported to be associated with different types of CDG. In our study, two patients (P14 and P15) had genetic variants in *COG5* (c.1039 C >T (p.Q347*), c.928 + 3 A >G) and *COG6* (c.1843 C >T (p.Q615*), c.428G >T(p.S143I)), respectively, the patient P15 had been reported before [32]. Their clinical phenotypes were shown in Table 1 and similar to previously reported cases.

**Fig. 5:**
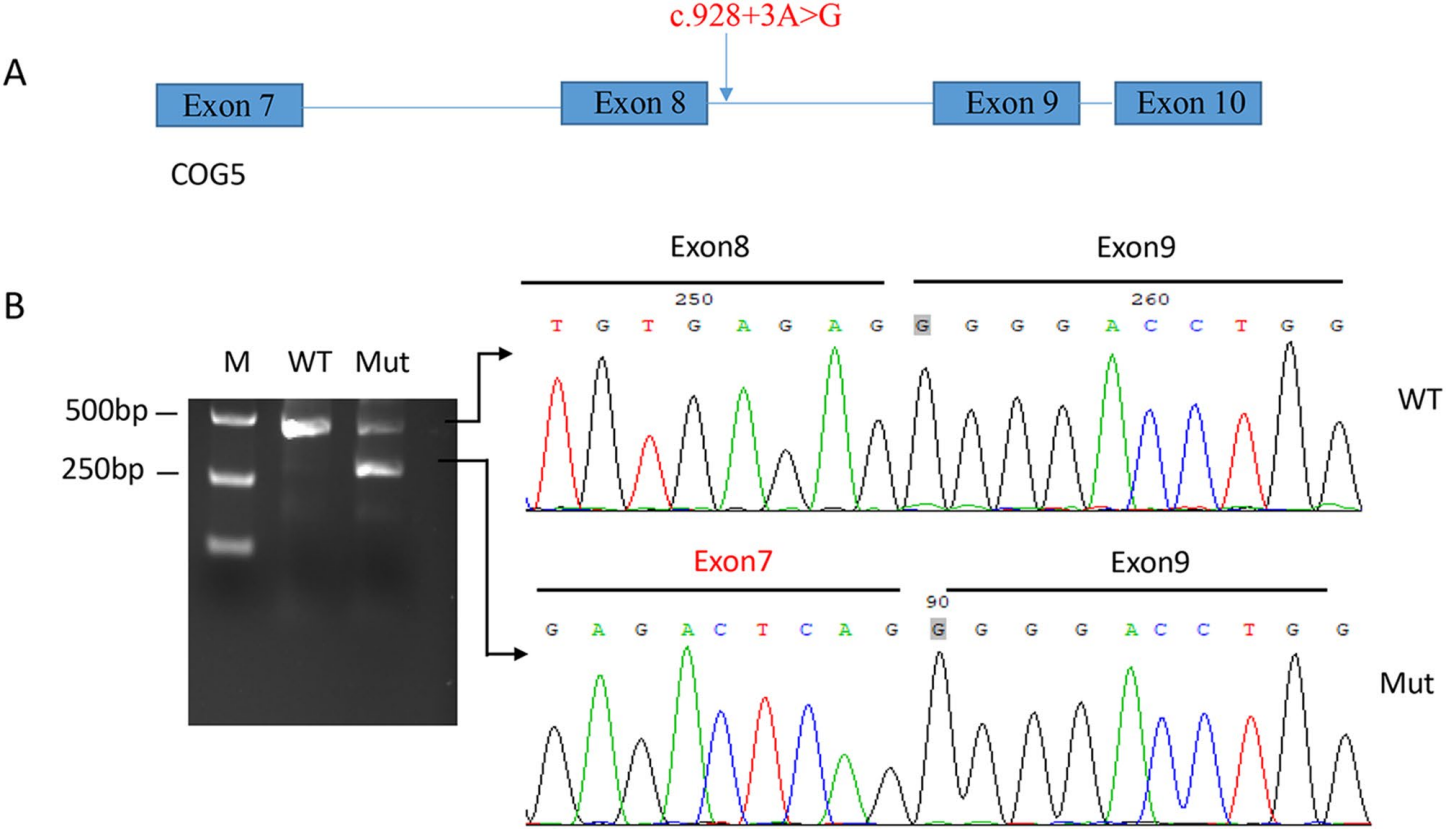
Minigene splicing assay analyses of COG5 variant c.928 + 3 A > G. (**A**) Pattern map of splice mutation location. (**B**) Agarose gel images of RT-PCR products for HEK293T cells and Sanger sequencing of the RT-PCR products from each clone. This variant resulted in a smaller amplifed fragment due to exon 8 skipping. M, DNA marker, WT, wild-type; Mut, mutation

Minigene splicing assay was performed to investigate the effect of the variant (c.928 + 3 A > G) in patient 14 on the splicing of *COG5*. Wild-type and variant plasmids were transfected in 293T cells and the result demonstrated that the splice variant (c.928 + 3 A > G) resulted in exon skipping (Fig. 5), and this variant was classified as LP according to the ACMG guidelines.

### Patients with variants in ALG1 gene

ALG1-CDG is an autosomal recessive disease with severe multiorgan involvement (OMIM 608540) caused by pathogenic variants in *ALG1* [33]. The most common phenotypic manifestations are developmental delay, intellectual disability, failure to thrive, hypotonia, and epilepsy. P16, 1-year-boy, had compound heterozygous variants in *ALG1* gene (NM_019109, c.328 C >A(p.Q110K), and c.863–2 A >G), and these two variants were classified as LP and P according to ACMG guidelines. This boy had drug-resistant epilepsy, facial dysmorphisms and abnormal liver function and died at 14 month old. Prenatal diagnosis was performed for this family when his mother was pregnant and they got a healthy baby.

## Discussion

Despite global recognition, CDG is under reported in China, with limited case studies available. This is first comprehensive report from China to analyze 20 diagnosed CDG cases, highlighting clinical manifestations such as seizures, motor delays, facial dysmorphism, and growth retardation, supported by findings like abnormal MRI neuroimaging (e.g., cerebellar atrophy), elevated CK levels, and abnormal liver function. This study underscores the importance of integrating genomic tools and systematic evaluations to improve CDG diagnosis and characterization.

A recent large cohort study of 280 individuals with genetic data consistent with a CDG diagnosis found that (s) developmental delay was the most frequent presenting symptom (77%), followed by hypotonia (56%) and feeding difficulties (42%) [34]. Similarly, in our cohort, development delay was the most common initial symptom, observed in all individuals by the time of enrollment, followed by facial dysmorphism (64.7%), abnormal liver function (52.6%), and seizures (50%). The broad differential diagnosis associated with these symptoms, reflecting significant clinical heterogeneity, continues to pose a challenge for timely CDG diagnosis.

We found 28 distinct variants, including 11 novel variants, in 13 different genes: *ALG1*, *ALG2*, *ALG3*, *ALG11*, *ALG13*, *COG5*, *COG6*, *MOGS*, *DPM2*, *DPM3*, *SSR4*, *SLC35A2*, and *PMM2*. The majority of variants were missense (78.5%), followed by nonsense (10.7%), splice site (7.1%), and frameshift deletions (3.6%) (Table 2). According to ACMG classification guidelines, 8 variants were classified as pathogenic, 14 as LP, and 6 as VUS. Autosomal recessive inheritance was observed in 80% (16/20) of cases and 20% (4/20) displayed X-linked inheritance.

The most common variants were in genes associated with disorders of N-linked protein glycosylation, including *PMM2* (3 patients from 3 families), *ALG2*(3 from 2 families), *ALG13* (2 patients), *ALG1* (1), *ALG3* (1), *ALG11* (1), *MOGS* (1), and *SSR4* (1), followed by genes causing disorders of dolichol metabolism (DPM2 and DPM3) and disorders of Golgi trafficking and transport (*COG5*, *COG6*, and *SLC35A2*), which is consistent with previous report [34]. This study significantly expands the current mutational spectrum of CDG, and also revealed considerable genetic heterogeneity, reflecting the complex molecular underpinnings of CDG.

In this study, we conducted a correlation analysis between genotype and phenotype by retrieving all previously published cases, including our patients, for these genes associated with uncommon CDGs.

*ALG2* variants could develop ALG2-CDG or ALG2-CMS, by reviewing literature, a total of 9 cases of ALG2-CMS and 5 cases of ALG2-CDG were reported from 7 articles [13, 14, 35–39]. Together with our study, 11 variants in *ALG2* have been identified. we noticed that variants located before the intramembrane region (N-terminal, before 158aa) are associated with ALG2-CMS, while variants occurring after the intramembrane region are linked to ALG2-CDG, which presents with more severe clinical symptoms (Fig. 3). ALG2 takes part in the fourth and fifth step of lipid-linked oligosaccharide synthesis, with two different mannosyltransferase activities. Specifically, Alg2 adds both α 1,3-and α1,6-mannose onto ManGlcNAc_2_–Pdol to form the trimannosyl core Man_3_GlcNAc_2_-PDol [30]. The Alg2 conserved C-terminal EX7E motif, the N-terminal cytosolic tail, and 3G-rich loop motifs, are essential for these enzymatic activities, both in vitro and in vivo [40]. Variants in these regions impair the enzyme’s function, which likely contributes to the more severe clinical manifestations observed in ALG2-CDG compared to the relatively milder phenotype of CMS caused by *ALG2* variants.

DPM2-CDG is an extremely rare form of CDG, and the main clinical manifestations of these patients include hypotonia, intractable epilepsy, muscle damage, elevated serum CK levels, and microcephaly [15]. To date, including our cases, only 7 cases from four families have been identified [15–17]. DPM2 contains two putative transmembrane domains (amino acid residues 11–31 and 49–69) [16]. Variants in the first domain, such as Phe21 and Tyr23 substitutions, impair its ability to bind with DPM1 [41], suggesting that the first transmembrane region is crucial for maintaining the stability of the complex. Our analysis suggested variants affecting the first transmembrane region or disrupting it were associated with more severe symptoms, and three patients with such variants died before the age of 3 years, in contrast, variants in the second transmembrane region resulted in less severe manifestations (Fig. 4A), however, further study with more cases is needed to confirm this correlation.

SSR4-CDG is ultra-rare X linked, comparably mild subtype of CDG, presenting mostly in males. We reported a 16-month-old boy, who had most of the key symptoms of SSR4-CDG, developmental delay, speech delay, cognitive impairment, feeding difficulties, and muscular hypotonia. In addition, this patient had febrile seizure and ocular abnormality. However, he had no obvious microcephaly, and his facial feature is not as typical as described previously, which could be due to the young age.

*SLC35A2*, located on chromosome Xp11.23, encodes UGT-1 and is part of the SLC35 family of nucleotide sugar transporters. *De novo* variants in this gene are associated with SLC35A2-CDG, often with epileptic encephalopathy as a prominent feature [20]. Somatic *SLC35A2* variants have also been reported in patients with focal seizures and suspected focal cortical dysplasia [21]. Furthermore, recent studies have linked brain somatic variations in *SLC35A2* to mild malformation of cortical development with oligo dendroglial hyperplasia in epilepsy [42]. While SLC35A2-CDG typically shows a strong gender bias, with most cases occurring in females, there have been reports of male patients with a milder phenotype, including minor neurological involvement and growth deficiency [23, 24]. By reviewing literature, we postulate that males with hemizygous variants in *SLC35A2* may present with a milder phenotype compared to those with mosaic variants in brain, although this requires further research. We slao found that the mosaic variants carried by male patients were mostly frameshift or nonsense, whereas all variants found in five male patients including our patient were missense. Loss of function variants may be tolerated in males when in the somatic state, but likely embryotic lethal in the germline, and this may be athe reason for gender bias in SLC35A2-CDG. Our study has some limitations. First, due to the small number of patients enrolled, we were unable to analyze more specific genotype-phenotype correlations. The rarity of CDG and the limited screening of this condition in China may partly explain this limitation. Second, changes in glycan composition in patient serum were not analyzed by MALDI-TOF MS due to the lack of necessary equipment. In addition, we identified some variants of VUS, functional studies were not conducted. Further research with larger patient cohorts and long-term follow-up would enhance our understanding of these diseases, which is critical for elucidating pathogenic mechanisms, improving the genotype-phenotype correlation, refining genetic counseling, and developing new treatments.

## Conclusion

We identified 28 disease-causing variants, including 11 novel variants, using WES in 20 CDG patients. Our findings expand the spectrum of known variants and their related clinical phenotypes in Chinese CDG patients and establish possible genotype-phenotype correlations of several genes.

## Data Availability

All data and materials are available from the corresponding on reasonable request.

## Abbreviations

CDG: Congenital disorders of glycosylation
VUS: Variants of uncertain significance
WES: Whole-exome sequencing
EEG: Electroencephalography
GATK: Genome Analysis Toolkit
BWA: Burrows-Wheeler Aligner
HPO: Human Phenotype Ontology
AST: Aspartate aminotransferase
ALT: Alanine aminotransferase
SNV: Single nucleotide variants
MAF: Minor allele frequency
